# Therapy of early breast cancer: current status and perspectives

**DOI:** 10.1007/s00404-025-08028-0

**Published:** 2025-04-22

**Authors:** Nikolas Tauber, Niklas Amann, Dominik Dannehl, Thomas M. Deutsch, Moritz Dimpfl, Peter Fasching, Andreas Hartkopf, Sabine Heublein, Lisbeth Hilmer, Manuel Hörner, Natalia Krawczyk, Annika Krückel, David Krug, Frederik Marmé, Laura L. Michel, Mattea Reinisch, Achim Rody, Henning Schäffler, Andreas Schneeweiss, David Utz, Kristina Veselinovic, Maggie Banys-Paluchowski

**Affiliations:** 1 Department of Obstetrics and Gynecology, University Hospital Schleswig-Holstein, Campus Luebeck, Luebeck, Germany; 2 Department of Obstetrics and Gynecology, Comprehensive Cancer Center Erlangen-EMN (CCC ER-EMN), Erlangen University Hospital, Friedrich Alexander University of Erlangen-Nuremberg (FAU), Erlangen, Germany; 3 Department of Obstetrics and Gynecology, University Hospital Tuebingen, 72016 Tuebingen, Germany; 4 Department of Obstetrics and Gynecology, University Hospital Heidelberg, Heidelberg, Germany; 5 Department of Obstetrics and Gynecology, Medical Faculty Mannheim, University Medical Center Mannheim, University of Heidelberg, Mannheim, Deutschland; 6 Department of Obstetrics and Gynecology, University Hospital Ulm, 89075 Ulm, Germany; 7 Department of Obstetrics and Gynecology, University Hospital Duesseldorf, 40225 Duesseldorf, Germany; 8 Department of Radiotherapy and Radiation Oncology, University Medical Center Hamburg-Eppendorf, Hamburg, Germany; 9 National Center for Tumor Diseases, University Hospital and German Cancer Research Center Heidelberg, 69120 Heidelberg, Germany; 10 German Cancer Research Center (DKFZ), 69120 Heidelberg, Germany; 11 Department of Internal Medicine VIII, Medical Oncology and Pneumology, University Hospital Tuebingen, 72016 Tuebingen, Germany

**Keywords:** Early breast cancer, Future perspectives, Surgical de-escalation, HER2-positive, Triple-negative, Hormone-receptor positive

## Abstract

Medical advancements in breast cancer are truly remarkable. Especially in recent years, numerous new therapeutics have been approved and surgical strategies have been de-escalated for specific patient groups. In the therapeutic setting, CDK4/6 inhibitors as oral maintenance therapy in early breast cancer and immune checkpoint inhibitors (Pembrolizumab) for triple-negative breast cancer (BC) are noteworthy. In the surgical field, prospective randomized controlled trials have currently explored the possibility to deescalate axillary surgery by omitting sentinel lymph node excision (INSEMA, SOUND). As a result, there have been significant improvements in prognosis and a reduction in surgical morbidity for patients. Many exciting trials are underway, and it remains to be seen whether antibody–drug conjugates beyond trastuzumab emtansine, will find their way into the treatment lines for early-stage BC. Furthermore, the integration of artificial intelligence in both diagnostics and treatment recommendation evaluation is a promising area with great potential.

## Introduction

Breast cancer is the most common malignant disease among women in the European Union, accounting for more than 400.000 new cases annually [1]. Statistically, one in eight women is diagnosed with breast cancer in her lifetime, with a median age at initial diagnosis of 65 years [2]. Survival outcomes are highly dependent on tumor stage and subtype. Early breast cancer, defined as a disease confined to the breast and/or axillary lymph nodes, is considered curable. Advances in multimodal therapies have improved the chances of achieving long-term cure to over 75% of patients [2]. Optimal management of breast cancer requires a multidisciplinary approach in specialized breast cancer units [3, 4]. Recently, the use of artificial intelligence in diagnostics, as demonstrated in the PRAIM study, has led to a significant increase in the detection rate of breast cancer within the mammography screening program, with an improvement of approximately 18% [5].

At the molecular level, breast cancer is a highly heterogeneous disease, characterized by distinct biological subtypes. Key molecular features include the overexpression of human epidermal growth factor receptor 2 (HER2) and the expression of hormone receptors. In daily clinical routine, surrogate intrinsic subtypes are defined using histologicaly and immunohistological analysis of proteins such as estrogen and progesterone receptors, HER2, tumor grading and the proliferation marker Ki67. These molecular features define the HER2-enriched, luminal-like and triple-negative breast cancer surrogate subtypes, which primarily guide the therapeutic strategy [6].

The interdisciplinary management of breast cancer integrates systemic and locoregional approaches, which have advanced remarkably over the past decade [7]. This article aims to provide a comprehensive update on current strategies and explore emerging perspectives in the management of early breast cancer.

## Surgical therapy

### Breast

There is a notable trend toward less radical surgical techniques in breast cancer surgery and related axillary procedures. Survival rates following breast-conserving surgery with adjunctive radiotherapy are comparable to, if not superior to those achieved with mastectomy [6, 8, 9].

### New marking techniques for non-palpable lesions

Recommended marking techniques for non-palpable lesions in breast-conserving surgery include well-established wire-guided localization or intraoperative sonographic localization [6, 10, 11]. Recent technological innovations have facilitated the development of further localization methods, such as magnetic and paramagnetic markers, radioactive seeds, radar reflectors and radiofrequency identification tags [6, 12]. All marking techniques are comparably effective. The choice of techniques depends on local conditions, the physician's experience, and technical requirements. The use of radioactive seeds is not permitted in some European countries due to potential radiation exposure. Additionally, the costs for marking and detection vary significantly among the different techniques. All localization techniques are currently being investigated in the MELODY study [13].

### Surgical margins

In cases of invasive breast cancer, ensuring tumor-free surgical margins is crucial (“no ink on tumor”), while for ductal carcinoma in situ, a minimum margin of 2 mm is advisable [14–16]. Provided that complete resection of all BC lesions is achievable, breast-conserving surgery is feasible for multifocal or multicentric lesions [6, 17]. A reliable marking of all suspicious lesions is fundamental prior performing a breast-conservating therapy in patients with multicentric or multifocal disease. For inflammatory breast cancer, mastectomy continues to be the endorsed surgical approach [6].

### Risk-reducing surgeries for genetic predisposition

Another cohort for whom mastectomy should be considered includes carriers of germline pathogenic variants (PVs) associated with an elevated risk of breast cancer. While prophylactic surgical measures are an option for patients, especially those with *gBRCA1/2* PVs, and have been shown to improve overall survival in younger patients [18], the benefit for other genetic PVs remains unclear. However, recommendations for risk-reducing surgery need to clearly differentiate between preventative strategies for healthy individuals and those already diagnosed with cancer. A recent study has shown for the first time that contralateral risk-reducing mastectomy (CRRM) provides an overall survival benefit for breast cancer patients up to the age of 40 years [18]. Recent ESMO (European Society for Medical Oncology) guidelines indicate that for women with stage I-III high-risk PV-associated breast cancer, excluding those with *TP53* PVs, breast-conserving therapy with adjuvant radiotherapy represents a viable and safe alternative to risk-reducing mastectomy (RRM) [19]. Consideration of RRM should be based on a comprehensive assessment encompassing disease prognosis, risks and benefits, patient preferences, the patient’s age, comorbidities, prior and prospective therapies, anatomical specifics, and the potential effects on sexuality and psychological well-being [19].

### Safety aspects of nipple-sparing mastectomy and skin-sparing mastectomy

Following the decision on mastectomy, it is crucial to carefully determine whether a nipple-sparing mastectomy (NSM) or skin-sparing mastectomy (SSM) can be implemented as these techniques often yield more favorable cosmetic results as opposed to modified radical mastectomy (MRM). Local recurrence rates are comparable between SSM and MRM. However, strict adherence to the contraindications for SSM, such as extensive skin involvement and inflammatory breast cancer, is recommended. Oncological safety is maintained in the preservation of the nipple-areola complex (NAC) when a complete resection with clear margins is achieved [6]. Tumor characteristics increasing the risk of NAC involvement and, therefore, a risk–benefit evaluation with the patient is crucial as second surgeries may follow. Risk factors for nipple involvement are a retroareolar or central tumor location, tumor-nipple distance under 2 cm, tumor size greater than 5 cm, multicentricity and the presence of lymph node metastasis or lymphovascular invasion. NAC infiltration in breast cancer is also thought to be influenced by tumor biology, with risk factors such as advanced tumor grade, positive human epidermal growth factor receptor-2 status, and negative estrogen and progesterone receptor status [20]. Patients with a recommendation for a mastectomy should always be counseled about breast reconstruction, particularly about possible timing (immediate vs. delayed) and type of reconstruction (implant-based vs. autologous) [6].

### Future perspectives of breast surgery

Recent literature reveals the potential of pharmacological interventions in the perioperative setting, notably through approaches like the preoperative peritumoral infiltration of local anesthetics, which has shown to significantly improve disease-free survival (DFS) and overall survival (OS) of patients with early breast cancer in one study from India [21]. Alongside a trend towards less aggressive surgical techniques, the future of breast surgery may also increasingly involve drug-based strategies to further improve patient outcomes.

### Axilla

The status of axillary lymph nodes is among the most critical prognostic factors in breast cancer, and axillary staging is an integral component of surgical management in the early stages of the disease [1]. Until the 1990s, systematic axillary lymph node dissection (ALND) was considered the standard of care for all patients with invasive breast cancer. However, over the past two decades, the extent of axillary surgery has been progressively reduced, and axillary surgery is currently primarily regarded as a diagnostic procedure to guide post-operative treatment decisions rather than a therapeutic intervention. The choice of surgical strategy depends on the pretherapeutic clinical lymph node status (cN0 vs. cN +), the planned type of breast surgery (breast-conserving surgery vs. mastectomy), and whether a primary operation or neoadjuvant therapy is being pursued [6].

### Primary surgical therapy

Since the early twenty-first century, sentinel lymph node biopsy (SLNB) has become the standard surgical approach for patients with clinically negative lymph nodes (cN0), replacing classical ALND in this context. This less invasive technique has significantly reduced postoperative morbidity. Simultaneously, SLNB has demonstrated comparable oncological safety in several prospective randomized trials [22]. For many years, the standard practice for cases where SLN involvement was confirmed intraoperatively was to proceed with the completion ALND, while cases of SLN negativity did not necessitate further surgical intervention. However, the management of patients undergoing upfront surgery changed after the ACOSOG Z0011 trial and current national and international guidelines discourage routine ALND in cases where 1–2 SLNs are metastatic [23]. The ACOSOG Z0011 trial demonstrated that completion ALND offers no additional benefit for patients treated with breast-conserving surgery and subsequent adjuvant radiation therapy. While the results of the ACOSOG Z0011 study were limited to patients undergoing breast-conserving surgery, the recently published data from the prospective, randomized, multicenter SENOMAC study demonstrated similar results in patients receiving a mastectomy [24]. The estimated 5-year recurrence-free survival was 89.7% (95% CI 87.5–91.9) in the SLNB-only arm and 88.7% (95% CI 86.3–91.1) in the ALND arm [24].

Patients with clinically suspicious lymph nodes scheduled to receive primary surgery are recommended ALND. In this context, it is worth noting that a preoperative minimally invasive biopsy of lymph nodes should be considered to confirm lymph node status and assess predictive markers.

### Axillary surgery in the neoadjuvant setting

In patients with cN0 breast cancer receiving neoadjuvant chemotherapy (NACT), SLNB after NACT is considered the standard surgical approach. This method enables comprehensive assessment of nodal response and reduces the need for ALND.

**Fig. 1:**
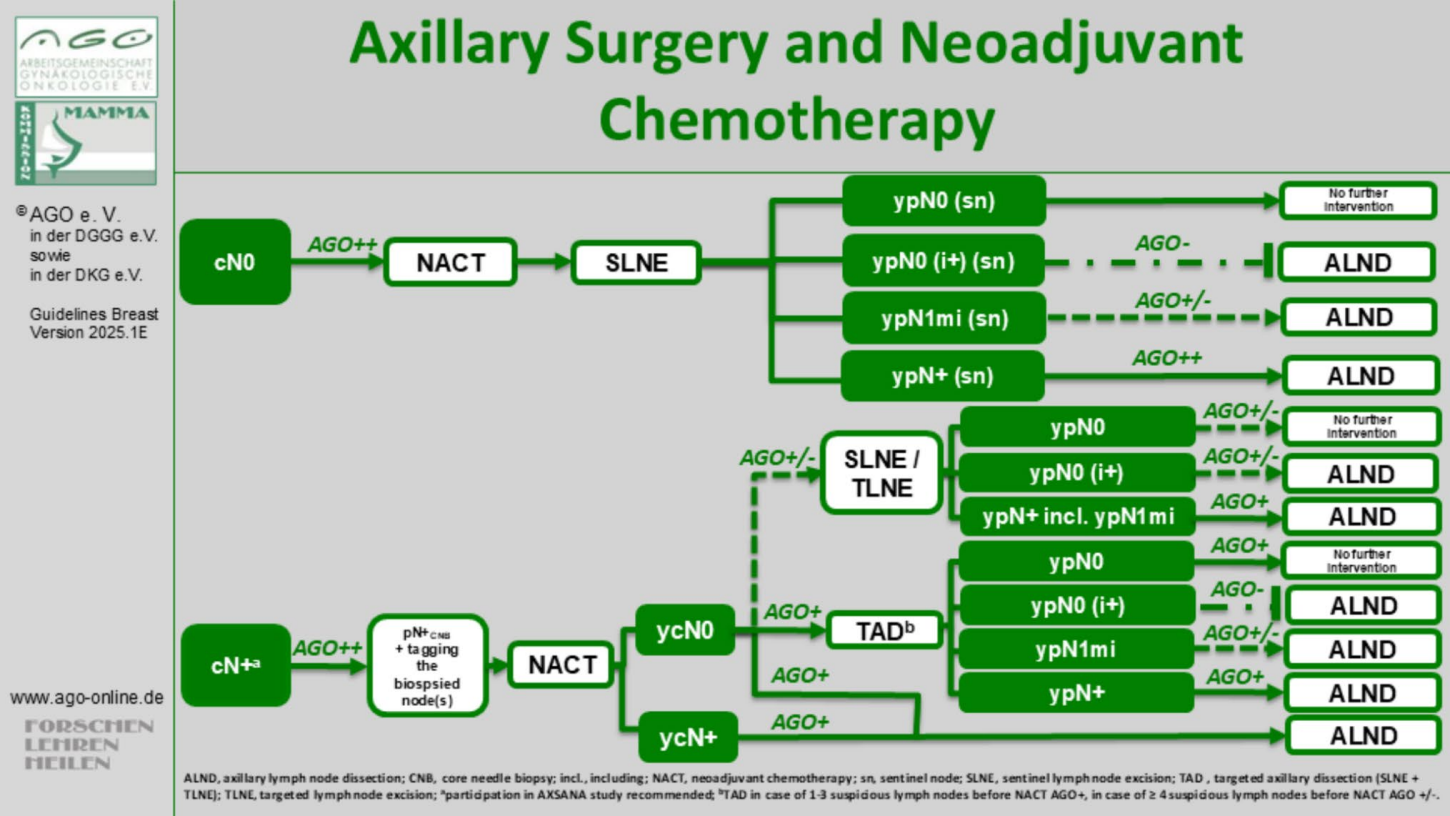
Therapy algorithm of the AGO Breast Committee for Axillary Surgery and Neoadjuvant Chemotherapy [6]

In patients initially presenting with clinically node-positive axilla who convert to clinically negative node status through NACT (cN + →ycN0), surgical de-escalation in the form of SLNB or targeted axillary dissection (TAD) is feasible [25]. The concept of TAD, introduced by Caudle et al. in 2015, involves marking one or more lymph node metastases before initiating NACT and removing both the marked target lymph node (TLN) and the SLN after NACT [26]. TAD was shown to reduce the false-negative rate of SLNB in this setting to less than 10% and is considered equivalent to ALND for patients with one to three (cN1) involved lymph nodes and clinical nodal remission (ycN0) [1, 22, 26]. Secondary ALND should be considered in case of SLN or TLN involvement after NACT due to the high likelihood of additional metastases [27]. Currently, it remains to be clarified whether patients with low residual disease (i.e., micrometastasis [AGO + / −]) benefit from completion ALND. For patients with isolated tumor cells, completion ALND is no longer recommended (AGO -) [28] (Fig. 1).

### Future perspective: is a complete omission of surgical axillary staging possible?

Ongoing studies explore the feasibility of omitting SLNB entirely in select cN0 patients. The recent results of the multicenter, randomized SOUND study showed no significant differences in five-year DFS (SLNB arm 94.7% vs. no surgery 93.9%) or OS (98.2% vs. 98.4%) between arms after a 5.7-year follow-up [29]. Similarly, the INSEMA trial (*n* = 5154) demonstrated that in breast cancers up to two cm, G1 and G2, cN0 patients 50 years and older, omitting SLNB was non-inferior to SLNB, with a five-year invasive DFS (iDFS) of 91.9% vs. 91.7% after six years of follow-up [30].

A complete omission of axillary staging will therefore likely become a safe option in the future not only for older patients [10] but also for selected patients over 50 years. The INSEMA study showed that refraining from SLNB did not compromise survival in patients with early-stage, cN0 breast cancer with low-risk disease (cT1, G1-G2, HR + HER2 −) receiving breast-conserving surgery [30]. The AGO Breast Committee therefore recommends the option of omitting SLNB (AGO +) for this patient cohort [1, 6].

## Radiotherapy in breast cancer

Radiotherapy, alongside pharmacological and surgical treatments, is one of the three main pillars in the treatment of early-stage breast cancer. The indication for radiotherapy and its specific modality depend primarily on the type of surgery performed and the postoperative pathological tumor classification. This section provides an overview of when radiotherapy is indicated for breast cancer treatment.

### Radiotherapy after breast-conserving surgery

**Fig. 2:**
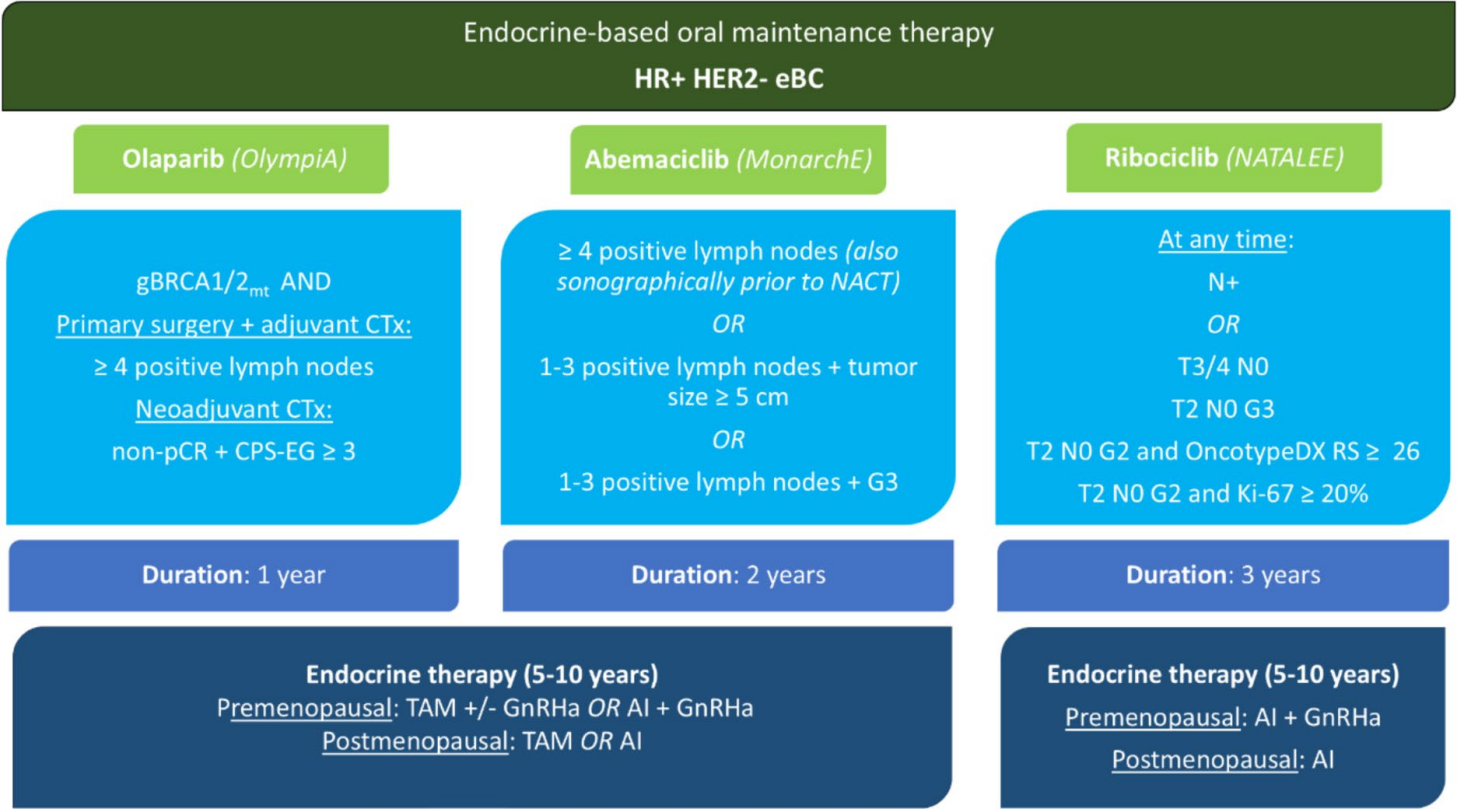
The key features of the three available strategies for the management of early HR + HER2 − breast [110]

Postoperative irradiation of the breast is typically performed using moderately hypofractionated percutaneous radiotherapy (fraction dose 2.5–2.67 Gy, total dose 40 Gy). Placement of clips in the tumor bed can enhance the precision of postoperative radiotherapy (e.g. with regard to the definition of radiation boost fields or planning of partial breast irradiation) [6]. Conventionally fractionated percutaneous radiotherapy (fraction dose 1.8–2.0 Gy, total dose up to 50 Gy) is no longer considered the standard after breast-conserving surgery. Preliminary data on ultrahypofractionation (fraction dose > 5 Gy) appear promising [31, 32] (Fig. 2). While the Royal College of Radiologists has declared ultra-hypofractionation as the standard of care, other guidelines have been more guarded [33].

An additional boost to the tumor bed should be considered based on individual risk factors (e.g., premenopausal status, and poor tumor differentiation). Although this approach has been shown to reduce local recurrence rates, no survival benefit has been demonstrated. For patients with low-risk tumors (e.g., pT1, pN0, G1-2, L0, HR + , non-lobular, age > 50 years, no BRCA-mutation known), partial-breast irradiation is an option for de-escalation. Data from non-randomized trials like LUMINA and IDEA suggest that radiotherapy may be omitted in early-stage luminal-A-like tumors (pT1 pN0) in patients aged 50–55 and older [34, 35], however, follow-up is limited to five years. Findings from the PRIME II trial demonstrated that local recurrence rates increased considerably with a follow-up of ten years when radiotherapy was omitted, especially in patients that discontinued endocrine therapy or had tumors with low ER-expression [35]. However, there was no difference in distant metastases or overall survival in patients who had no adjuvant radiotherapy.

### Postmastectomy radiotherapy (PMRT)

After mastectomy, there is no general indication for radiotherapy. However, if postoperative risk factors such as involvement of ≥ four axillary lymph nodes, tumor size pT3/4, or inflammatory breast cancer are present, postmastectomy radiotherapy to the chest wall is recommended. Recently presented results of the SUPREMO-trial suggest that PMRT does not improve prognosis in patients with intermediate-risk breast cancer. This trial mostly included patients with pT1-2 and 0–2 involved lymph nodes. However, all patients with involved lymph nodes underwent ALND and no data according to biologic subtypes were presented.

In cases of locally advanced breast cancer postmastectomy radiotherapy to the chest wall and lymphatic drainage pathways is indicated regardless of remission status following neoadjuvant chemotherapy [36]. Postmastectomy radiotherapy should administered using moderate hypofractionation. Recent trials demonstrated that moderate hypofractiation is also effective and safe in patients who underwent breast reconstruction [37, 38].

### Regional nodal irradiation

The ACOSOG Z0011 and AMAROS trials have significantly influenced axillary management in recent years [23, 39]. Both trials demonstrated that ALND can be safely omitted in patients with clinically node-negative breast cancer with SLN involvement. While radiotherapy treatment details were only published for a subset of patients in ACOSOG Z0011 and revealed that about half of patients received therapeutic doses to axillary level I-II and about 20% had supra/-infraclavicular fields (level III-IV), all patients in the radiotherapy-arm of AMAROS received therapeutic irradiation to level I-IV. Both trials included 30–40% of patients with micrometastatic lymph node involvement. In the recently published SENOMAC trial that only enrolled patients with macrometastatic SLN involvement, about 95% of patients had radiotherapy which mostly included regional nodal irradiation (RNI) to level I-IV [40]. The majority of patients in ALND-omission trials including AMAROS, OTOASOR and SENOMAC received RNI to level I-IV while only a minority had limited treatment to level I-II. The only randomized trial comparing these two approaches is the OPTIMAL trial [41]. The trial was stopped early due to slow accrual. Non-inferiority of limited RNI to level I-II could not be demonstrated, although outcome was favorable among patients on both arms.

RNI to level III-IV and the internal mammary region was demonstrated to improve overall survival in the recently published EBCTCG-meta-analysis [42]. The benefit was largest in patients with 4 or more involved lymph nodes, leading to universal guideline recommendation for this subgroup, while RNI would only be recommended in patients with 1–3 involved lymph nodes in the presence of additional risk factors such as medial/central tumor location and/or negative hormone receptor status.

### Radiotherapy after neoadjuvant therapy

Response to NACT adds additional prognostic information. Tailoring adjuvant radiotherapy based on response to NACT has been studied based on numerous retrospective studies. Data from the randomized controlled NSABP B-51 trial have been presented at SABCS 2023 [43]. This trial enrolled patients with cT1-3 cN1 disease that converted to ypN0 after NACT. In the case of BCS, patients were randomized to whole-breast irradiation with or without RNI. After mastectomy, PMRT with RNI was compared to the complete omission of adjuvant radiotherapy. At 10 years, there was no benefit in the extended radiotherapy arm. Some questions still remain unanswered since 80% of patients had a pCR in the breast and only a few patients had cT3-tumors. However, this trial suggests that de-escalation of PMRT and RNI may be possible in patients with nodal conversion after NACT.

A clear indication for regional nodal irradiation is given when there is residual axillary disease [36].

There is very limited data for exclusive RNI, thus RNI should be combined with chest wall/breast irradiation.

### Future perspectives

In the future, it will remain exciting to what extent the de-escalation of radiotherapy will be continued. Ongoing trials analyze the role of gene expression assays and other biomarkers for tailoring adjuvant radiotherapy [44]. Furthermore, neoadjuvant radiotherapy is studied in prospective trials such as Neorad. It is hoped that prospective trials such as AXSANA [45] and Alliance A11202 will provide further insight on surgical therapy and radiotherapy of the axilla after NACT [46].

## Systemic therapy

### HER2-positive breast cancer

The HER2 receptor is overexpressed in approximately 15% of breast cancers (DAKO score 3 + or 2 + with CISH/ISH [(chromogenic in situ hybridization/ in situ hybridization] positivity) [47]. HER2 overexpression is associated with a more aggressive tumor biology. Prior to the approval of HER2-directed therapy, HER2 overexpression was therefore associated with a poor outcome [48]. Today, HER2 positivity is associated with favorable outcomes due to advancements in HER2-targeted therapies, which began with the FDA (U.S. Food and Drug Administration) approval of the HER2-directed monoclonal antibody trastuzumab in 1998 [49, 50].

**Fig. 3:**
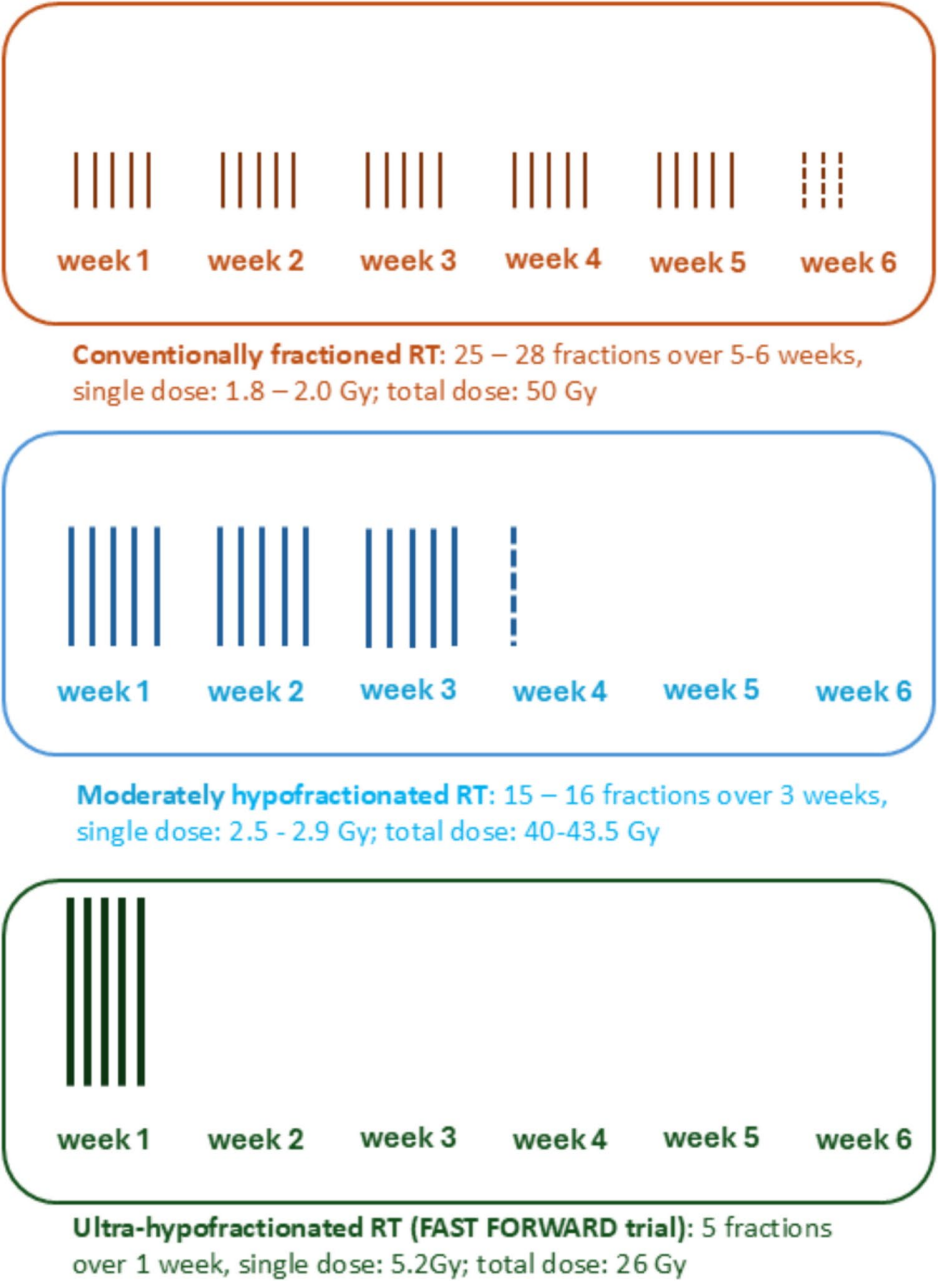
Presentation of the most common radiotherapy regimes for breast cancer

For tumors > 2 cm and/or node-positive disease, neoadjuvant HER2 blockade with trastuzumab and pertuzumab, combined with chemotherapy, is considered the standard of care [51]. The AGO Breast Committee recommends a combined multiagent chemotherapy regimen with trastuzumab and pertuzumab over six to eight cycles [52, 53]. Anthracycline-free regimens, such as TCHP (docetaxel, carboplatin, trastuzumab, and pertuzumab for 6 cycles), reduce cardiotoxicity compared to anthracycline-containing protocols [49]. In the case of pCR, trastuzumab and pertuzumab- if initially node-positive- are completed for one year [52] (Fig. 3).

### De-escalation strategies

For patients with low tumor load (cT1 cN0), primary surgery is advised [54, 55]. If postoperative findings confirm pT1 and pN0 status, a de-escalated adjuvant regimen of paclitaxel and trastuzumab for 12 weeks should be offered, maintaining trastuzumab for one year (Fig. 3).

### Escalation strategies

For patients who did not achieve pCR after neoadjuvant chemotherapy, a switch to the antibody–drug conjugate (ADC) T-DM1 is recommended for 14 cycles. The KATHERINE trial demonstrated that T-DM1 improved absolute iDFS by 13.7% and OS by 4.7% at 7 years (hazard ratio [HR] 0.66) [53]. In HR + HER2 + breast cancer, extended therapy with neratinib, an irreversible pan-HER2 inhibitor, improves iDFS at 5 years by 5.1% (HR 0.58) and OS at 8 years by 2.1% (HR 0.79) following trastuzumab-based adjuvant therapy [56] (Fig. 3).

### Future perspectives

New treatment strategies include both escalation and de-escalation and explore modern therapeutic approaches such as chemotherapy-free regimens, ADCs, and immunotherapy combinations [57].

The phase II PHERGain study demonstrated that patients who initially responded to HER2-targeted therapy alone can achieve pCR rates of 37.9% with a chemotherapy-free regimen [58]. Numerous other studies continue to pursue this approach such as the WSG-ADAPT umbrella trials. The WSG-ADAPT-HER2 + /HR- trial identified promising immune response signatures predictive of pCR [59]. Recently, the TOUCH trial showed that in HR + HER2 positive early breast cancer (eBC), endocrine combination therapy with palbociclib and dual blockade can achieve pCR rates comparable to those of paclitaxel plus dual blockade [60].

Other approaches aim to expand or replace conventional chemotherapy with new targeted substances like ADCs and immunotherapies. Approved in 2020, trastuzumab deruxtecan (T-DXd) features a higher cytotoxic payload (increased drug-to-antibody ratio) and a bystander effect, enabling action on neighboring cells with low HER2 expression. The phase III DESTINY-Breast11 trial also aims in this direction by investigating whether treatment with T-DXd, alone or in combination with trastuzumab, pertuzumab, and paclitaxel, can achieve better treatment outcomes compared to standard therapy [61]. In patients with high recurrence risk post-neoadjuvant therapy, the DESTINY-Breast05 trial compares T-DM1 with T-DXd. Results from the 3-year iDFS analysis are anticipated in Q3 2025 [62]. The ADAPT-HER2-IV trial stratifies HER2-positive patients according to their individualized risk profile, offering twelve to 18 weeks of neoadjuvant T-DXd versus conventional regimen [63].

The use of immunotherapy in early HER2-positive breast cancer is an emerging area of research with promising results. The phase II neoHIP trial demonstrated an 18.9% increase in the pCR rate when pembrolizumab was added to neoadjuvant taxane therapy and dual blockade [64]. The phase II DTP trial demonstrated that tumor-infiltrating lymphocytes (TILs), programmed death-ligand 1 (PD-L1) combined positive score (CPS) and HER2DX® luminal signature positively correlate with pCR in a chemotherapy-free regimen of durvalumab, trastuzumab, and pertuzumab and achieved a pCR rate of 67.6% [65].

### Triple-negative breast cancer

Early triple-negative breast cancer (TNBC) represents a challenging breast cancer subtype due to its aggressive nature and lack of hormone receptors or HER2 overexpression, which excludes targeted therapies like endocrine therapy or HER2-targeted agents. Consequently, the management of early TNBC relies heavily on chemotherapy and emerging immunotherapeutic options [66]. Treatment strategies are determined by the tumor stage at presentation, lymph node involvement, and the response to neoadjuvant therapy. Below, we summarize current evidence-based approaches and recommendations for TNBC treatment [1, 67].

### Adjuvant or neoadjuvant setting: what to use when?

**Fig. 4:**
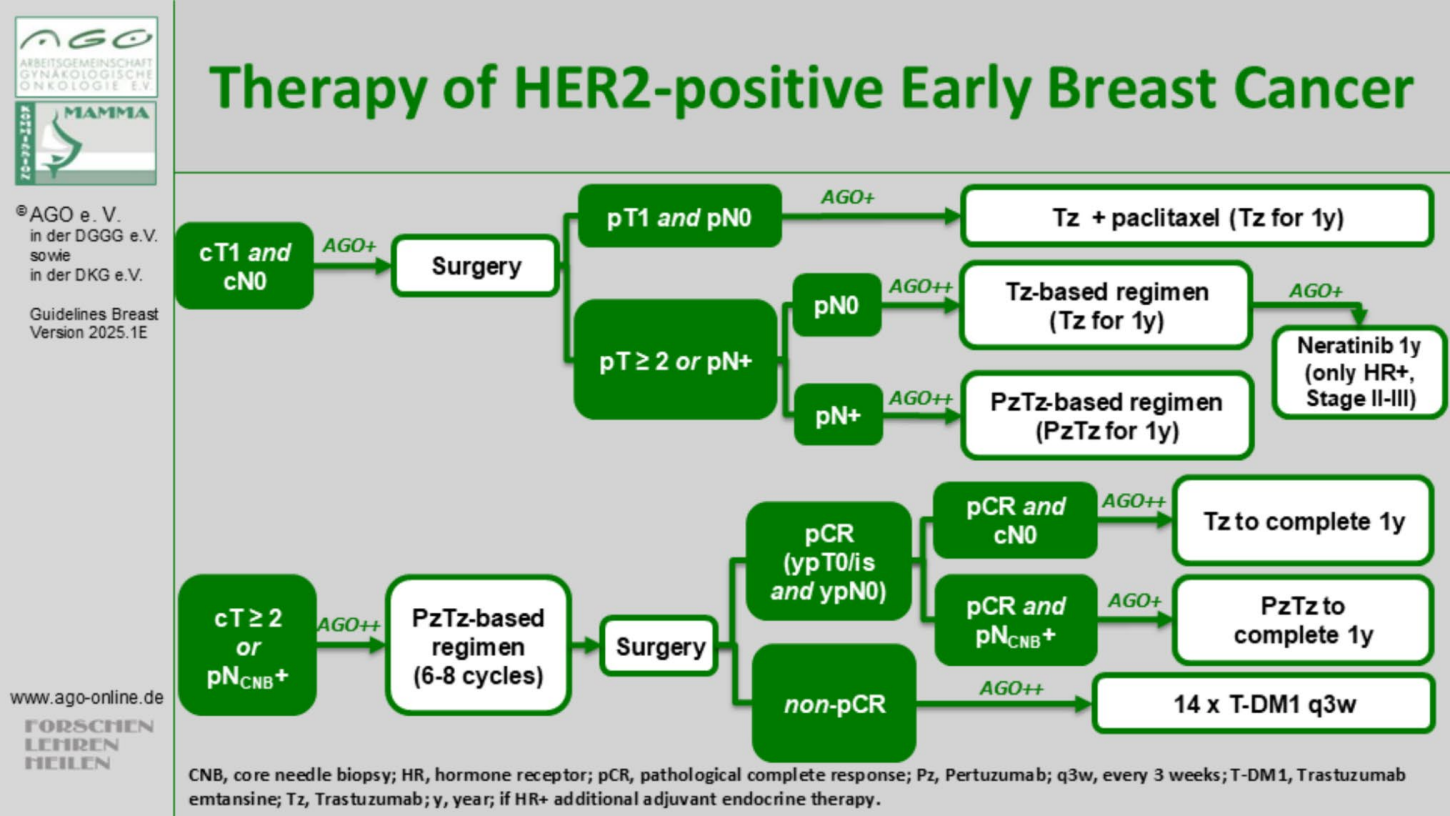
Therapy algorithm of the AGO Breast Committee for: Therapy of HER2-positive Early Breast Cancer [6]

For patients with small TNBC (≤ 5 mm, pT1a) without lymph node involvement, initial surgical resection without adjuvant chemotherapy is the standard approach. However, for tumors measuring > five to ten mm (pT1b), treatment options include either neoadjuvant chemotherapy followed by surgery or surgery followed by adjuvant chemotherapy [68]. These decisions are individualized based on patient-specific factors and tumor characteristics. In patients with larger tumors (> 1 cm), neoadjuvant chemotherapy employing an anthracycline-taxane sequence remains the standard of care (Fig. 4).

Chemotherapy regimens in the adjuvant and neoadjuvant setting are highly comparable and mainly consist of anthracycline-taxane-based regimens [68]. The incorporation of carboplatin into chemotherapy regimens has shown variable benefits depending on the treatment context. In the adjuvant setting, evidence supporting the addition of carboplatin is limited. In the neoadjuvant setting, carboplatin has been widely adopted as part of anthracycline-taxane-based regimens, particularly for stage II–III TNBC as shown by a recent meta-analysis [69].

### Immunotherapies for high-risk tumors

For patients with tumors larger than 2 cm or node-positive disease, the KEYNOTE-522 study showed that the integration of the immune checkpoint inhibitor pembrolizumab with anthracycline, taxane and platinum-based neoadjuvant chemotherapy improves iDFS and OS, irrespective of PD-L1 status [70]. Patients treated with pembrolizumab in the neoadjuvant setting are recommended to continue pembrolizumab for nine additional cycles post-surgery (Fig. 4). Due to the nature of the Keynote-522 study, it is not known whether pembrolizumab in patients with pCR are needed in the postneoadjuvant setting. Further trial are currently recruiting evaluating these questions [71].

### Post-neoadjuvant setting

Germline BRCA1/2 (gBRCA1/2) PVs are predictive biomarkers for patients with TNBC, offering the possibility of a targeted therapeutic option. The prevalence varies widely depending on tumor biology, disease stage, sex, age, familial predisposition, geographic location, and ethnicity [72]. In early-stage TNBC, the prevalence of *gBRCA1/2* PVs ranges from 6.5% to 15.4% [73]. Patients harboring these PVs generally exhibit greater chemosensitivity compared to those without *BRCA1/2* PVs, irrespective of the inclusion of carboplatin in their treatment regimen [74]. The landmark OlympiA trial demonstrated that PARP (poly(ADP-ribose) polymerase) inhibitor olaparib significantly improves distant recurrence-free survival and OS in patients with high-risk TNBC. Olaparib is indicated for TNBC patients with non-PCR after neoadjuvant chemotherapy or, tumors > 2 cm and/or nodal involvement if chemotherapy is given in the adjuvant setting [75]. Another option in the post-neoadjuvant setting is capecitabine, which has shown efficacy in patients with residual disease following neoadjuvant anthracycline and taxane chemotherapy, as evidenced by the CREATE-X trial [76]. Despite these therapeutic advancements, notable gaps persist in understanding the optimal combinations and sequencing of therapies for TNBC. For example, the efficacy of combining pembrolizumab with either capecitabine or olaparib in the post-neoadjuvant setting remain unexplored (Fig. 4).

### Future perspectives

The therapeutic landscape of early TNBC has advanced considerably with the introduction of immunotherapy and targeted approaches, particularly for BRCA-mutated tumors. Despite these advancements, chemotherapy remains the cornerstone of treatment. Continued research is essential to optimize existing post-neoadjuvant treatment protocols, explore the therapeutic potential of emerging treatment modalities such as antibody–drug conjugates, and refine combinatorial strategies involving current medications to improve clinical outcomes in this aggressive breast cancer subtype.

### Hormone receptor-positive HER2 negative breast cancer

HR + HER2 − tumors account for approximately 70% of eBC cases [77, 78]. Despite therapeutic advancements [79, 80] this subtype remains heterogeneous, with recurrence risks ranging widely [81, 82]. Notably, HR + HER2 − eBC is characterized by a persistent risk of late recurrences, with a 7-year recurrence rate of 7.2% in node-negative (N0) patients and up to 43.7% in N2-3 patients [82]. Therefore, effective management demands personalized risk assessment to minimize overtreatment and undertreatment.

### Standard treatments and risk stratification

**Fig. 5:**
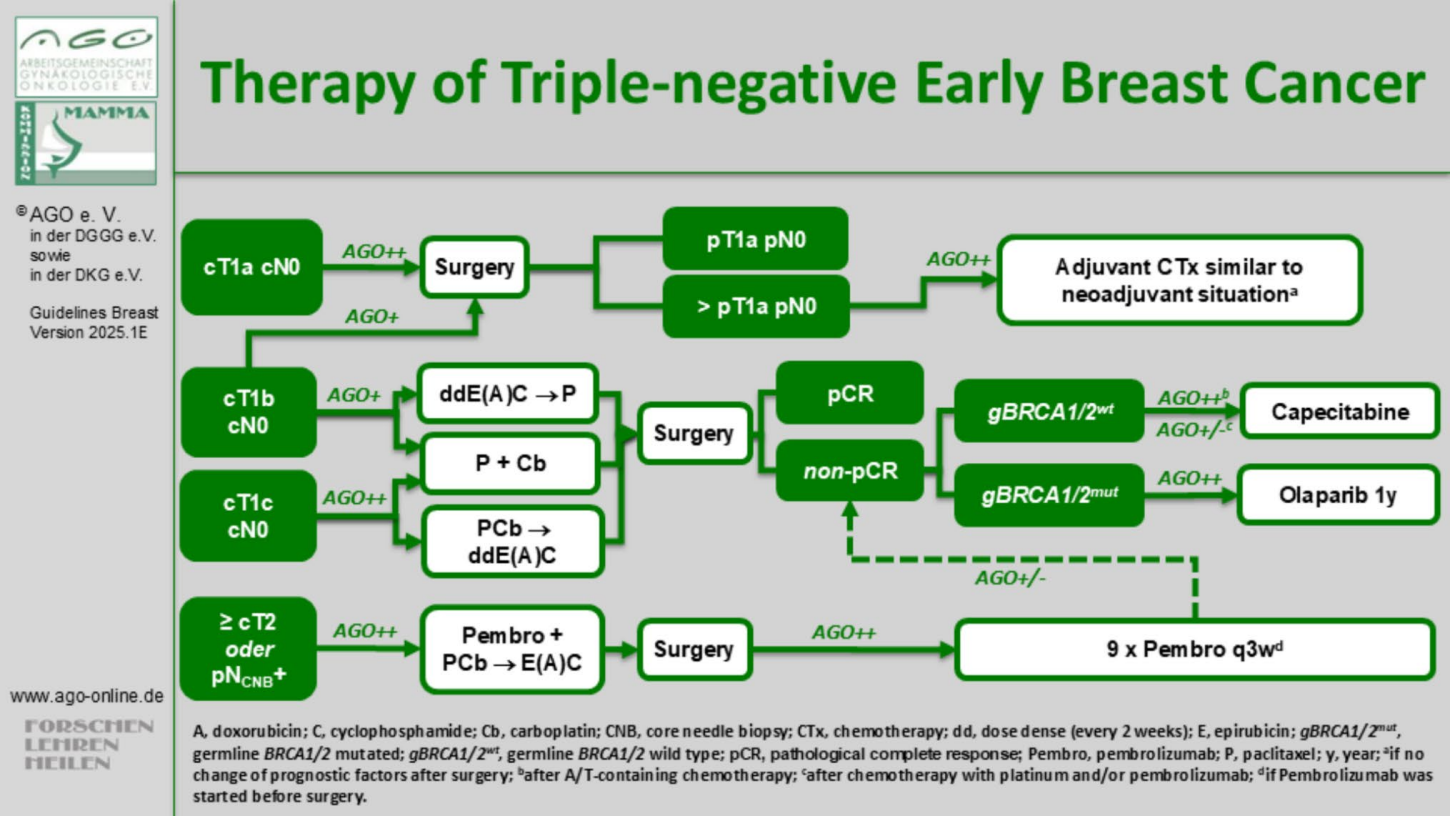
Therapy algorithm of the AGO Breast Committee for triple-negative early breast cancer [6]

Standard systemic therapy includes at least 5 years of endocrine therapy (ET). Additional therapies—(neo)adjuvant chemotherapy, extended ET, CDK4/6 inhibitors or PARP inhibitors—are tailored based on tumor stage, biology and menopausal status. Risk assessment remains challenging due to the absence of universal thresholds in guidelines [6, 67, 83–86]. Gene expression profiling is recommended in ambiguous cases, guiding chemotherapy decisions for patients most likely to benefit [1, 67, 83, 87] (Fig. 5).

### Gene expression assays

Prospective trials such as TAILORx [88], RxPONDER, [89] and MINDACT [90] confirm that gene expression assays such as OncotypeDX® and MammaPrint® can guide treatment decisions regarding the need for chemotherapy. While the available studies provide robust evidence for postmenopausal women with zero to three affected lymph nodes, premenopausal patients often derive greater benefit from chemotherapy, though the role of ovarian function suppression (OFS) remains uncertain [91].

### Ki-67 and endocrine response

Endocrine response, defined as a Ki-67 reduction to < 10% following a short-term (two to four weeks) endocrine induction, can further aid in refining risk stratification. The ADAPT study showed that chemotherapy omission is feasible in patients with 0–3 affected nodes, intermediate OncotypeDX Recurrence Scores (RS 12–25), and endocrine response [92]. Similarly, the POETIC trial found that postmenopausal women achieving Ki-67 ≤ 10% after two weeks of preoperative ET had reduced five-year recurrence risks, independent of clinicopathological factors [93].

### Chemotherapy

Dose-dense anthracycline/taxane regimens are preferred for patients requiring chemotherapy [94]. Non-anthracycline regimens may be suitable for intermediate-risk patients or those unable to tolerate anthracyclines [95–97].

### Endocrine therapy

Adjuvant ET for at least five years remains standard, with extended duration recommended for patients with higher recurrence risks [98]. Postmenopausal women preferentially receive either aromatase inhibitors (AI) in the first five years or sequential therapy, i.e., AI for two to three years followed by tamoxifen (TAM), or TAM followed by AI [81]. In patients with high-risk tumors, a total duration of seven to eight years is recommended [6]. Premenopausal women at low risk are generally treated with TAM [6], while higher-risk patients receive OFS [99] combined with TAM or AI [100]. Extending ET with TAM for an additional five years after an initial five-year course of TAM or AI should be considered in case of a higher risk of relapse [101, 102]. Switching to better tolerated ET is preferable to discontinuation. Adjuvant ET should be recommended even in cases of low estrogen receptor (ER) expression (1–10%) [103]. Concurrent bisphosphonates may prevent osteoporosis and improve outcomes [104].

### Combined endocrine-based therapy

**Fig. 6:**
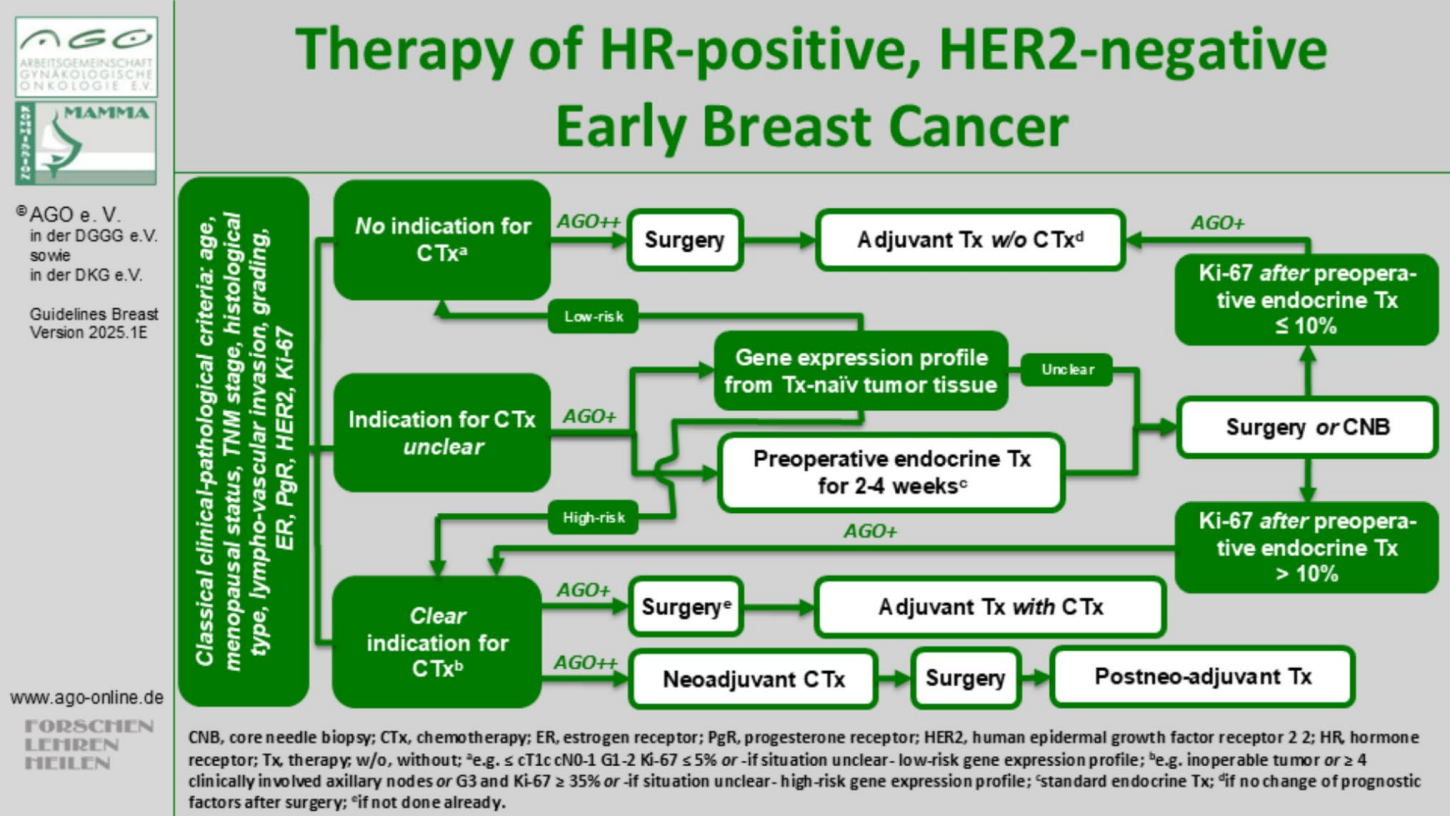
Therapy algorithm of the AGO Breast Committee for HR + HER2- early breast cancer [6]

Recent trials have expanded adjuvant therapy options for intermediate-to-high-risk HR + HER2 − eBC (Fig. 6):

- MonarchE: Two years of abemaciclib (150 mg twice daily) with ET improved four-year iDFS by 6.0% (HR 0.68, 95% CI 0.60–0.77) in patients with ≥ 4 positive lymph nodes or 1–3 nodes plus high-risk features (T3 or G3 tumors) [105].
- NATALEE: Three years of ribociclib (400 mg daily for three weeks followed by one week off) with ET improved four-year iDFS by 4.9% (HR 0.72, 95% CI 0.61–0.84) in high- and intermediate-risk patients, including node-negative cases with additional risk factors [106].
- OlympiA: One year of olaparib (300 mg twice daily) with ET significantly improved four-year iDFS by 7.3% (HR 0.63, 95% CI 0.50–0.78) and OS by 3.4% (HR 0.68, 95% CI 0.47–0.97) in g*BRCA1/2*-mutated, high-risk cases [62].

Real-world data indicate that over 30% of patients with HR + HER2 − eBC may be eligible for at least one of these options [107–111], which is expected to further improve outcomes.

### Future perspectives

Despite therapeutic progress, recurrence risks in HR + HER2 − eBC remain substantial. Further improvements hinge on recognizing subtype heterogeneity, developing novel biomarkers, and tailoring therapy to individual risk profiles. Emerging treatments options, including selective estrogen receptor degraders (SERDs; trials: ELEGANT, EMBER-4, LidERA, CAMBRIA 1/2 [112]) and immunotherapies (KEYNOTE-756 [113], I-SPY2 [114], CheckMate FTL7 [115]), are under investigation. The SURVIVE study will investigate biomarker-guided follow-up for intermediate-to-high recurrence risk [116], with related trials assessing interventions at the molecular recurrence stage [117]. A major challenge in the future will be to select the most effective therapy option for each individual patient, given the multitude of new therapeutic strategies. There is a high need for predictive biomarkers and the use of artificial intelligence will be particularly exciting in this context [118, 119].

## Conclusions

Breast cancer remains a leading cause of cancer-related death in women and a significant global health challenge. While new targeted therapeutic strategies emerge, a major focus of current trials is de-escalation in both locoregional and systemic therapy.

In the surgical field, new localization methods for tumor and lymph nodes have been introduced over the last years with the aim to reduce the extent of surgery and improve long-term health-related quality of life. Current studies aim to define patient groups with favorable tumor characteristics in which surgical interventions, such as axillary staging, can be completely omitted to spare selected patients unnecessary morbidity. De-escalation is also a common theme among radiotherapy for breast cancer, where a more targeted delivery, better patient selection and risk stratification as well as techniques such as partial breast irradiation have led to a more tailored and individualized approach.

Regarding systemic therapy, an emerging trend is the shift away from untargeted chemotherapy toward personalized and tumor-specific treatments. This approach now includes a wide range of strategies such as immune checkpoint inhibitors, PARP inhibitors, antibodies, ADCs, and combined endocrine-based regimens). As the formation of dedicated oncological centers increases and the number of doctor-patient interactions in outpatient clinics rises, there is a growing trend toward oral therapies for certain tumor settings. This trend is exemplified by the recent adoption of combined endocrine-based therapies for HR + HER2 − eBC.

Although many therapeutics, such as novel ADCs, are currently limited to metastatic setting, medical history shows that new treatment options usually prove their efficacy in these advanced stages first before being introduced into the (neo-)adjuvant treatment of earlier tumor stages. The future challenge lies in identifying the optimal treatment path for each patient from the multitude of therapeutic options, while not only prolonging life but also preserving quality of life through improved clinical management of new side effects.

## Data Availability

No datasets were generated or analysed during the current study.

## Abbreviations

ADC: Antibody–drug conjugate
AGO: Arbeitsgemeinschaft Gynäkologische Onkologie
AI: Aromatase inhibitors
ALND: Axillary lymph node dissection
CDK4/6: Cyclin-dependent kinases 4 and 6
CISH: Chromogenic in situ hybridization
CPS: Combined positive score
DFS: Disease-free survival
eBC: Early breast cancer
ER: Estrogen receptor
ESMO: European Society for Medical Oncology
ET: Endocrine therapy
FDA: U.S. Food and Drug Administration
HER2: Human epidermal growth factor receptor 2
HER2 +: Human epidermal growth factor receptor 2 positive
HER2-: Human epidermal growth factor receptor 2 negative
HR: Hazard ratio
HR +: Hormone receptor positive
HR-: Hormone receptor negative
iDFS: Invasive disease-free survival
ISH: In situ hybridization
MRM: Modified radical mastectomy
NAC: Nipple-areola complex
NACT: Neoadjuvant chemotherapy
NSM: Nipple-sparing mastectomy
OFS: Ovarian function suppression
OS: Overall survival
PARP: Poly(ADP-ribose) polymerase
pCR: Pathological complete response
PD-L1: Programmed death-ligand 1
PMRT: Post-mastectomy radiotherapy
PV: Pathogenic variants
RNI: Regional nodal irradiation
RRM: Risk-reducing mastectomy
SLNB: Sentinel lymph node biopsy
SSM: Skin-sparing mastectomy
TAD: Targeted axillary dissection
TAM: Tamoxifen
T-DM1: Trastuzumab emtansine
T-DXd: Trastuzumab deruxtecan
TILs: Tumor-infiltrating lymphocytes
TLN: Target lymph node
TNBC: Triple-negative breast cancer
